# Patients and public are important stakeholders in health technology assessment but the level of involvement is low – a call to action

**DOI:** 10.1186/s40900-020-00248-9

**Published:** 2021-01-05

**Authors:** Janet L. Wale, Samuel Thomas, Dominique Hamerlijnck, Ronald Hollander

**Affiliations:** 1 HTAi Patient and Citizen Involvement in HTA Interest Group (PCIG), 11A Lydia Street, Brunswick, Victoria 3056 Australia; 2 Avalere Health, 1201 New York Ave, NW, Suite 1000, Washington, DC 20005 USA; 3 Patient Expert European and Dutch Lung Foundation, EUPATI Fellow, HTAi PCIG Member, Zeeburgerkade 540, 1019HR Amsterdam, Netherlands; 4 INCA International Neuroendocrine Cancer Alliance, Newton, Boston, MA 02461, USA

**Keywords:** Health technology assessment, HTA, Patient and public involvement, Multi-stakeholder, Science of participation, Communication and partnership, Evidence-informed, Deliberative process, Transformative partnership, Patient leadership

## Abstract

**Background:**

Health technology assessment (HTA) agencies have an important role in the evaluation and approval of new technologies. They determine their value within a health system so to promote equitable, quality care with available healthcare resources. Many HTA agencies have some mechanism for involving patients in their processes, but there is great variability and an absence of comprehensive, robust practices for involvement. The accelerating pace of medical innovation creates a need to improve the depth and breadth of patient involvement in the HTA process.

**Main body:**

In this ‘Call to action’, we present ideas from three HTA expert commentaries calling for collaborative learning and to share innovative ideas for changes in HTA. We also draw on examples of HTA agencies creatively pursuing this goal. We propose a ‘Call to action’ for HTA stakeholders to undertake serious dialogue with patient advocates aimed at creating shared goals. HTA agencies can use these goals to ensure meaningful patient involvement at every step of the HTA process. Five elements are explored. In ‘Recognizing the value of shared purpose’, we highlight examples of HTA agencies that have patients working in partnership with medical practitioners and HTA staff. Results include improved processes that instil confidence. ‘Committing to patient involvement as part of HTA culture’ highlights several initiatives aimed at changes in HTA organisational culture to be more inclusive of patients. In ‘Aligning patient and HTA goals’ we cite work in Belgium and New Zealand which places a greater emphasis on quality of life rather than life expectancy and cost-effectiveness. By ‘Integrating patient involvement at every step of the HTA process’ patients can make vital contributions at every stage of the HTA process. We provide two examples of where HTA agencies have successfully involved patients early in the process in order to broaden the scope of evaluations. ‘Developing a common language and working together’ can support transformative dialogue through ‘unified language’.

**Conclusion:**

The authors of this commentary ask that agencies and stakeholders involved in HTA take up this call to work together for visionary and transformative elevation of the voice of patients in HTA worldwide.

## Plain language summary

As present, millions of people around the globe are receiving vaccinations for the Covid-19 virus. These vaccines were developed in months, not years, and on entirely new platforms. This is just the latest example of the accelerating pace of science and medical innovation. Health technology assessment (HTA) agencies play a vital role in assessing the safety, efficacy, cost and benefit of new treatments. Their decisions can have profound impact on the length and quality of patients’ lives.

This daunting task demands the greatest care; not just to evaluate the technical and financial dimensions of a new treatment but to carefully assess the full impact on patients. New treatments not only impact patients’ health status, they can profoundly impact quality of life. Treatments often come with requirements, side effects, and daily living burdens that can only be fully appreciated from the perspective of patients’ ‘lived experience’.

HTA agencies use various approaches to incorporate patient input in their work. A wide variety of patients and HTA practitioners came together to discuss these efforts during a 2019 HTAi workshop. While several promising examples of patient involvement were examined, the consensus of the patient leaders in attendance was that much more needs to be done. Following that workshop, the authors did additional research and continued the conversation with colleagues. This article reviews some promising initiatives. It proposes a ‘Call to action’ for collaboration among HTA agencies and stakeholders to develop a robust framework for patient involvement at all stages of the HTA process.

## Background

### About health technology assessment

Health technology assessment (HTA) is a systematic evaluation of a health technology [1]. Health technologies include drugs, diagnostic tests, medical devices and healthcare procedures. HTA addresses the questions: does the technology in question work? For whom does it work? How well does it work? At what cost does it work? How does it compare with other technologies that are already being used? HTA provides a scientifically-based methodology for the introduction of new interventions and the effective use of resources in health care. It sets out to determine the direct, intended effects of a technology and any indirect or unintended consequences [1]. HTA agencies are the organisations that set up these evidence-based assessment programs [2]. Health Technology Assessment international (HTAi) is a global, non-profit, scientific and professional society for people who produce, use or have an interest in HTA. HTAi Interest Groups, such as the Patient and Citizen Involvement in HTA Interest Group (PCIG), share international experiences and expertise. Each year HTAi has an annual meeting where Interest Groups and others run a scientific program that includes workshops [3].

### Workshop at HTAi annual meeting

In 2019, the PCIG workshop was entitled, “Listen, Exactly what do we want from community engagement in HTA”. HTA patient and public involvement coordinators, researchers, industry and patient advocates participated. During the workshop, discussions among the patient advocates led to a proposal for a ‘Call to action’. This requests that ‘all key stakeholders work together to create a clear visionary goal for meaningful patient involvement at every step of the HTA process; that ways of integrating patient experience and providing context are developed across a broad, diverse range of the population of a country or region’. A patient has ‘lived experience’ of a disease or condition and its treatment within the context of the healthcare system. In our ‘call to action’ we ask for greater involvement of patients across HTA, including in topic selection, evidence development, assessment, deliberations leading to recommendations, or decision making, on funding, subsidies or reimbursement. Funding mechanism can be through insurance plans or government (e.g. [4]).

### About HTA decision making

HTA agencies are responsible for discussions, or deliberations, on the value of a technology. Deliberation is indeed a useful way of teasing out and interpreting evidence and determining matters of value [2]. This discussion leads to recommendations or decisions on funding, subsidy or reimbursement of costs, and therefore access by patients to new technologies. Decision making is complex as the assessment of overall value of a technology may vary depending on the perspective taken, the stakeholders involved, and the context in which decisions are made. Patients and their carers are important stakeholders in the HTA process as they are the recipients of the technologies [5]. A stakeholder is any group or individual who can affect or is affected by the decisions made by the HTA agencies [6]. Included are industry representatives, clinicians, patients, families and carers, payers and policy makers. Members of the public, as taxpayers, have an interest in a sustainable and effective health system that uses public funds wisely [7].

The term HTA practitioner is used to describe HTA researchers and others who conduct HTAs or use the findings to inform policy making [1]. Stages of the HTA process include topic selection, scoping of the topic, assessment or evaluation of the clinical and cost effectiveness of the technology, and appraisal. A multi-stakeholder committee deliberates on the evidence and the values of each stakeholder to formulate their recommendations or decisions. These are then communicated, often with a right to appeal [8, 9].

#### Morally or ethically defensible decision making

HTA agencies generally work under the principles of fairness and legitimacy or reasonableness. To do this, they use consistent, well-defined processes for assessment and apply scientific rigour in the evaluation of the evidence [5]. Then follows deliberations on value among a diverse group of people including ‘public’ representatives [10]. Costs and the effectiveness of the technology can be important considerations for agencies [10].

#### Governance and structure

Agencies vary in how they are structured and their governance, including how close they are to government as the payers of health services. The National Institute for Health and Care Excellence (NICE) in England was set up to have a level of separation from political and medical profession drivers [2]. NICE states that its role is to improve outcomes for people using the NHS and other public health and social care services [4]. Legally NICE, for example, must recover the cost of the HTA from the company that expects to market drugs in England [4]. Canadian Agency for Drugs and Technology in Health (CADTH) is an independent, not-for-profit organization established by the federal, provincial, and territorial Canadian governments. Its vision is that Canada has a world-class system for assessing and managing health technologies to achieve better outcomes and value for Canadians. This is achieved by delivering credible scientific evidence and management strategies for the use of the technologies [11]. Other agencies are situated within government but with independent appraisal committees. In Australia the chair of the Pharmaceutical Benefits Advisory Committee (PBAC) is independent [12] and in Singapore this is a ministerial position [13]. Overall, HTA agencies have a variety of governance structures. This adds to the complexity of recommending best practices that fit multiple agencies in different countries.

### Patient input into HTAs

Patients and patient groups have roles in some parts of HTAs in a number of countries including the United Kingdom, Canada and Australia among others [14]. This is not a systematic involvement from beginning to end of the HTA process, and a number of countries are progressing their processes for patient involvement and finding challenges [15]. NICE has recently outlined where and which patients are involved in their HTA processes, with examples of impact. Roles include in scoping the topic for assessment, providing patient experts to answer questions during the deliberative process, inviting patient groups to provide templated submissions to inform assessment and the appraisal committee, and in developing guidance on use [9]. CADTH asks patient groups to provide templated submissions when the assessment process is being formulated, at protocol stage. The submissions also inform appraisal committee deliberations [16]. For Scottish Medicines Consortium (SMC) meetings, patient groups present their own submissions during committee deliberations and answer any questions [17]. The practices to involve patients at other HTA agencies worldwide range from no involvement to involvement in select phases of the HTA process.

### Relating ‘HTA expert commentaries’ to a ‘Call to action’

The publication of a survey of international HTA agencies to better understand how HTA agencies are using deliberative processes, and the need for guidance [8] was followed by three commentaries by people well known in HTA [2, 5, 10]. The viewpoints expressed in these commentaries resonate with the proposed patient advocate ‘Call to action’ for greater patient involvement in HTA. The commentaries call for innovative thinking in HTA around possible improvements in HTA processes to improve accountability.

In the present article we define goals for the ‘call to action’. Under each goal we describe points raised in the commentaries (as Points from ‘HTA expert commentaries’) and other publications. We also highlight examples of what has been done – to demonstrate the how, what, where and why of building on patient involvement in HTA.

### Goals of the ‘Call to action’ are

1. To work together with shared purpose. 2. A change in HTA culture with integrated patient involvement. 3. Alignment with HTA agency goals, and a positive impact on the diverse populations served. 4. Patient involvement at every step of the HTA process. 5. Transformative thinking that involves patient leaders, with use of a unifying language.

#### ‘Call to action’

That all key HTA stakeholders, including patients and carers, work together to create a clear visionary goal for meaningful patient involvement at every step of the HTA process. That ways of integrating patient experience and providing context are developed across a broad, and diverse range of the population of a country or region.

Some of the values and practicalities associated with ‘meaningful’ involvement in research have been discussed in a pragmatic way, for example that people feel valued, respected and see their involvement makes a difference [18].

#### Goal 1. To work together with shared purpose

Patients have a right to participate in the planning and delivery of their healthcare, and it follows that they have a right to be part of the HTA process [19]. The various members of the HTAi PCIG worked together to develop ‘Values and Standards for patient involvement in HTA’ [20]. The values are as follows. ‘Fairness’ is where patients have the same rights to contribute to the HTA process as other stakeholders, and where HTA processes enable effective engagement. Patients have unique knowledge, perspectives and experiences that can contribute to essential evidence for HTAs, and therefore ‘relevance’. There is ‘equity’ by seeking to understand the needs of a diverse population of patients. ‘Legitimacy’ is provided by patients participating in HTA processes and contributing to the transparency, consistency, accountability and credibility of decision making. ‘Capacity building’ is needed in order to address the barriers to patients and HTA agencies working together.

Shared purpose and purposeful involvement with defined goals are two of the quality criteria developed by the multi-stakeholder group ‘Patient Focused Medicine Development’ for patient engagement in drug development [21]. Other quality criteria are respect for the resources and energy required for providing patient input, representativeness of stakeholders with agreed roles and responsibilities, the resources and capabilities to enable meaningful engagement, transparency in communication and documentation, and continuity and sustainability.

### Points from ‘HTA expert commentaries’

The evidence-informed deliberative appraisal processes undertaken by HTA committees are important to ensure decisions made are relevant and take into account viewpoints from a diverse group of stakeholders when determining value [8]. It is important to include patients and carers as ‘major’ stakeholders [5]. Schlander [10] expressed the importance of closing the existing gap between the narrowly defined assessment reports, based for example on clinical trials for product regulation, and the broader deliberations by HTA appraisal committees to assess value. Evidence should be presented in a way that facilitates these subsequent deliberations.

### Examples of where changes are happening

Patients, medical practitioners and HTA staff have been shown to work effectively together, to provide input into deliberations, in the Patient and Clinical Expert (PACE) meetings of the SMC [22]. Technology sponsors can request a PACE meeting for an orphan or ultra-orphan and end of life medicine. A summary of the meeting ispresented at the SMC committee meeting alongside the patient group submissions. Wale and Sullivan [16] found that the PACE input was taken very seriously and integrated throughout the ‘committee discussion report’.

Another example is with Institut national d’excellence en santé et en services sociaux (INESSS) in Quebec, which utilises joint discussions between patients and medical practitioners as part of its HTA processes [23, 24]. Patients could contribute with their lived knowledge of a health condition; contribute to decisions in summarising the literature, and to address concerns of patients. Expert patient committee perspectives were incorporated into the final HTA report, in particular relating to quality of life, healthcare decision-making experiences, and best practices in the decision making [23].

Roy [25] also showed that patients, users of healthcare services and carers can provide experiential and contextual knowledge to inform discussions on provision of hospital services. They describe ‘real people and their lived experience’ as real data that can contribute to every stage of assessment and appraisal. Such data would better equip decision makers in their decisions. The authors used a three-round Delphi process to co-construct an engagement policy for the healthcare and social services technology assessment unit of the Centre intégré universitaire de santé et services sociaux de l’Estrie - Centre hospitalier universitaire de Sherbrooke (CIUSSS de l’Estrie-CHUS) in Quebec, Canada. They argue that including experiential and contextual knowledge leads to greater legitimacy or defensibility of HTA recommendations. This is achieved by addressing fair process with appropriate selection and diversification of participants in the discussions, management of conflicts of interest using relevant criteria and evidence for decision making with different worldviews represented.

Under the first goal, we have highlighted values and quality criteria for working together with shared purpose. We give examples of patients with experiential and contextual knowledge working in partnership with medical practitioners and HTA staff. These have led to development of processes for defensible decision making in HTA and in providing patient input that contributes to HTA appraisal and decision making.

#### Goal 2. A change in HTA culture with integrated patient involvement

HTA agencies aim to apply rigorous, impartial methodologies that involve rules of practice, hierarchies of evidence, and clearly defined definitive outcomes, often with strict cost-effectiveness criteria [10]. This is to deliver best evidence using accepted methodological standards that are objective, credible and trustworthy to enable the appropriate use of health technologies (e.g. [11, 13]). Focus groups and a survey of eight HTA agencies from Europe and the Americas highlighted that objective quantitative data was seen to provide scientific rigour for assessment reports [26].

At the same time, patients’ expectations of health care are increasing as innovative and more personalised treatments are becoming available; and more attention is being given to patient reported outcomes [27, 28] and in person-centred health service delivery [29]. Studies of patient involvement in HTA have identified limitations in the use of patient group input as templated submissions [16], as expert patients at committee meetings [30] and in reporting on the use of the input from patient groups [17].

### Points from ‘HTA expert commentaries’

Here the commentary was mainly in the form of questions. What would be needed for HTA agencies to reflect on the deeper meaning of scientific rigour, and adjust their practices as needed? [5] Could rigorous integration of qualitative research, including structured consultation of patients, citizens and relevant stakeholders, analyses of the socio-political and the organisational contexts, identification of ethical aspects across all the dimensions of evaluation, contribute to a better assessment? [5]. Goetghebeur and Cellier [5] stated that there is a need for transparency in which methods were used, which data was selected, and which arguments, elaborated by whom, were used to generate recommendations for public funding or subsidies. Involvement of relevant stakeholders in the discussions or deliberations of HTA committees can be seen to lead to decision making and recommendations that are fair and able to be defended with logic and justification; thus making them trustworthy. These authors [5] questioned from an HTA perspective if the HTA process covers all relevant aspects for fair and reasonable decision making, and if all relevant stakeholders are involved. They also questioned what mechanisms can be put in place to make sure this happens? Furthermore, what is the effectiveness of the HTA process in addressing the issues or concerns of decision-makers, patients and public as the ‘chief interested parties’? What role is given to public and patients as ‘chief interested parties’? [5].

Two of the commentaries asked if the information made public from HTA deliberations is helpful, sufficient and necessary. This is for justifying funding and informing all stakeholders, including patients [5, 10].

### Examples of where changes are happening

HTA agency attitudes to patient involvement are slowly changing [31, 32]. Boothe looked at the Ontario Committee to Evaluate Drugs and the pan-Canadian Common Drug Review, now part of CADTH. She used surveys and interviews over a number of years to describe how the different players in HTA appraisals saw the goals of patient involvement, and how thinking had changed over time. More dialogue is now possible with technical experts on the goals, strengths and challenges of patient involvement in Canada [31]. The Belgian Health Care Knowledge Center (KCE) in a recent study [32] used a Board Game to assess the attitudes and culture of its employees toward patient involvement. Quoted texts are informative: “..someone needs to take up the leadership for the patient involvement activities and to coordinate the activities with sufficient resources and training for both researchers and patients”; “Empowering relationships and partnerships with patients, and developing mutual trust are important for meaningful patient involvement and participation”).

Under the second goal, the ‘expert commentaries’ raised many questions that are important to address on how patients could be involved and why, to set the path for a ‘change in culture’. We have highlighted examples of discussions that have been initiated to address changes in culture of HTA agencies to be more inclusive of patients.

#### Goal 3. Alignment with HTA agency goals – and a positive impact on the diverse populations served

An important consideration for patients is the impact and value of HTA agencies for the populations served. Agencies want to demonstrate their impact in terms of policy decisions and changes in clinical practice, but where both HTA processes and individual HTA findings could be better disseminated [15].

##### Stated goals (visions or missions) of HTA agencies

NICE states that its role is to improve outcomes for people using the National Health Service (NHS) and other public health and social care services [4]. The vision of CADTH is that Canada has a world-class system for assessing and managing health technologies to achieve better outcomes and value for Canadians, with appropriate use of health technologies [11]. The Agency for Care Effectiveness (ACE) in Singapore’s vision is to improve patient outcomes and healthcare value through HTA, with objective and credible healthcare guidance to enable stakeholders to optimize health benefits within finite resources [13]. European Network for Health Technology Assessment (EUnetHTA) states that it supports evidence-based, sustainable and equitable choices in healthcare and health technologies [33]. In Thailand, the Health Intervention and Technology Assessment Program (HITAP) vision is a state of the world in which the only health interventions and technologies available at public expense are all demonstrably effective and available for all, and so innovate excellent HTA with social impact [34].

This shows an emphasis on methodologies and better health outcomes across the population through equitable provision of health technologies.

### Points from ‘HTA expert commentaries’

In their commentary, Goetgebeur and Cellier [5] argued that decision making should reflect the mission and values of the HTA agency. And decision making should be fair and reasonable. This includes the data considered, the stakeholders involved, and the methods and processes by which these are selected and organised. Further research is needed to understand how the present processes could support these objectives, and to stimulate an international reflection on what works best and what needs to be adjusted.

The HTA process starts with reasoned prioritisation of the interventions for assessment, or topic selection. An independent, consultative approach could contribute to the creation of overall value for patients and the population served [5], rather than existing funding models for some HTA agencies (e.g. [4]) and commissioning models [15]. Furthermore, Schlander [10] highlighted that systematically leaving out patients with rare and ultra-rare disorders would not fit with the increasingly well-documented wish of citizens to share resources in a way which does not exclude patients who require services that are not ‘efficient’, that is, cost-effective.

### Examples of where changes are happening

Two countries, Belgium (KCE commissioned by the public payer National Institute for Health Insurance (RIZIV/INAMI)) and New Zealand (Pharmaceutical Management Agency or PHARMAC) have in recent years engaged with their public to change their processes for funded access or subsidies [35]. Both countries now have new funding processes that place emphasis on quality of life rather than cost-effectiveness, and the separation of individual versus societal perspectives.

Both countries wanted to explicitly account for public preferences, better align decision-making processes with public values, needs and preferences, and validate or legitimise final HTA decisions. At the same time, the public consultation processes provided an opportunity for people to learn about the payer’s operations, how funded access decisions are made, and what is considered [35]. Transparency of processes is an important part of gaining trust and legitimising HTA activities [36]. The outcomes of Belgium’s process were an emphasis on quality of life rather than life expectancy, integrating patient experiences with scientific evidence in the HTA process, and involving multidisciplinary teams, with a plea for solidarity [32]. Solidarity can be described as fellowship arising from common responsibilities and interests.

This third goal, alignment with HTA agency goals, provides an emphasis on methodologies and better health outcomes across the population through equitable provision of health technologies.

Belgium and New Zealand have updated their HTA processes. They now place a greater emphasis on quality of life and of separation of individual patients from the broad population, rather than life expectancy and cost-effectiveness across all diseases. This can be seen as a move to achieve better health outcomes and therefore the stated goals of a number of HTA agencies, along with equitable distribution of technologies. It is important for accountability that agencies are able to demonstrate their impact on health systems and the value they add.

#### Goal 4. Patient involvement at every step of the HTA process

For a fair and reasonable approach to HTA, this goal proposes that patients should be involved in HTAs right from the beginning through to completion. Patients can be involved in prioritising topics for assessment, informing literature searches, critically appraising evidence, taking part in appraisal committee deliberations as stakeholders and ensuring patient and carer experience and context is part of the assessment and in formulating recommendations.

In some countries, for example CADTH in Canada, members of the public (Common Drug Review [37]) or patients (pan-Canadian Oncology Drug Review [38]) are represented on the appraisal committees. Some appraisal committees also seek input from patients and patient groups on patient experiences of a health condition and its treatment (e.g. [9, 16]). Appraisal committees that seek patient input use it to identify knowledge gaps, for example on outcomes which, if improved, would significantly change the experience of living with a health condition [39]. Although the rationale for patient and public involvement is clear [19, 40], overall progress in involvement of patients in HTA globally has been challenging [14, 15]. O’Rourke et al. [15] highlighted ten challenges for HTA agencies that included lack of human resources, time restrictions, limitations in funding and challenges in designing an effective and efficient process for engaging stakeholders, in particular patients and clinicians.

##### When HTA appraisal committees discuss the supporting evidence, it can be important that the design of clinical trials involved patient advocates

A challenge identified by O’Rourke [15] was to evolve existing HTA methods and processes – associated with a greater need for incorporating observational and real world data. When HTA agencies assess and then discuss clinical evidence in appraisal committees, inclusion of patients in the design of clinical trials could play a pivotal role. Aspects of clinical trials that patient advocates could influence are determination of clinical outcomes and inclusion of patient-reported outcomes [27], and other parameters such as the populations studied, duration of studies, monitoring, and follow up [28].

### Points from ‘HTA expert commentaries’

The role given to patients and the public should be clear, as well as the drivers of the evolution of HTA processes [5]. Furthermore, reasoned prioritisation of technologies on which to do HTAs, involving multiple stakeholders, can create value for patients and the populations served [5]. Use of fair and reasonable processes for appraisal committee deliberations implies the collection of ‘factual information’, competing arguments and different viewpoints that are then integrated within the societal context of the HTA agency. Yet we need to examine what is the ‘best’ or most appropriate evidence for different HTA decisions. And no broadly accepted, ethically defensible frameworks guide fair and reasonable deliberations and decision making [10].

#### Examples of where changes are happening

Some HTA systems involve patients late in the process, and in the form of a testimony or submission to an appraisal committee [17]. HTA researchers make decisions on what is taken from clinical trials for consideration in HTA appraisals and economic evaluation. The KCE report [32] identified that patients can help in the scoping of projects to better describe the context of the research topic and take patient issues into account. They can assist with selection of patient relevant outcomes for consideration in an assessment, as well as decisions about the recruitment of study participants and therefore the informative nature of the evidence. The selection and testing of the data collection instruments, and defining minimal important differences in outcomes are other areas that they can have influence. For transparency, the patient contributions and their potential impact on the research process should be reported in the research report [32].

Like KCE, researchers at Institut national d’excellence en santé et en services sociaux (INESSS), Quebec, identified that it would have been good for patients to meet INESSS researchers earlier to include more qualitative research in their literature search and review; and for advice to be sought from expert patients [23].

Under the fourth goal of patient involvement at every step of the HTA process, we have highlighted that patients can have a role in HTA processes from topic selection, scoping, examining the evidence, the deliberations of appraisal committees to determine value and in developing recommendations or decisions. We have provided two examples of where HTA agencies have successfully utilised patients early in the process in order to broaden the scope of a report or evaluation. Early involvement in the design of clinical trials that are to provide clinical evidence on new technologies is also important, to ensure that patient relevant information is available. Under the fourth goal of patient involvement at every step of the HTA process, we have highlighted that patients can have a role in HTA processes from topic selection, scoping, examining the evidence, the deliberations of appraisal committees to determine value and in developing recommendations or decisions. We have provided two examples of where HTA agencies have successfully utilised patients early in the process in order to broaden the scope of a report or evaluation. Early involvement in the design of clinical trials that are to provide clinical evidence on new technologies is also important, to ensure that patient relevant information is available. We need to examine what is the ‘best or most appropriate evidence’ for different HTA decisions.

#### Goal 5. Transformative thinking that involves patient leaders – with use of a unifying language

HTA agencies are part of a healthcare system. Many health systems are placing an emphasis on person-centred care and shared decision making between patient and healthcare provider [29]. Patient involvement has already become an important consideration in planning, design, service delivery, quality improvement, and systems transformation of health services [41]. In formulating the fifth goal we have therefore looked at discussions beyond HTA. We ask that HTA staff, practitioners and stakeholders work together with patient advocates to re-examine the objectives, roles and current tools and processes for patient involvement in HTA. For example, existing tools and how they are used for patient input may limit the usefulness of some input, for both patients and the HTA agencies organisations [17]. And integrating objective evidence with the findings from the lived experience as provided by patients and their carers remains a challenge, but can still contribute to the relevance of HTA recommendations [9]. The experiences of patients and carers together with the accumulated knowledge of healthcare professionals can provide valid sources of evidence if empowered to do so [42]. ‘Patient leadership’ could help systems understand how to invest in active, meaningful patient engagement and involvement. Working together could be transformative. We can explore that, A. Lived experience can be valid evidence within the right parameters and with a willingness to do so. B. Concepts such as ‘patient leadership’ can help systems understand how to invest in active, meaningful patient engagement and involvement. C. A unifying language could help, and seeing ‘participation as a science’ might legitimise patient involvement in the eyes of HTA practitioners. D. Capturing everyday experiences from a global environment can help to throw light on problems and lead to guidance on how to achieve identified goals of patient involvement.McNally [43] used the term ‘patient leadership’ to describe an investment in patient and carer leaders working collaboratively in co-design projects around patient feedback. Similarly Pomey [44] described a process of ‘patient leadership’ for patient involvement within a health service in Québec. This commentary illustrates how the language used in HTA can be challenging. Palmer [42] argues that unifying language is needed where patients are to work as partners, and is part of a movement for a ‘science of participation’. Gathering and sharing of experiences to gain greater understanding of the issues, and an evidence base of impact and outcomes can be built through co-production. In the process, HTA practitioners can explore and learn about how patients and public communities engage with and interpret the knowledge and evidence base for assessment of new technologies. Experiences could vary from country to country, and training and skills building are important for people from diverse backgrounds to encourage new and innovative approaches [45].

### Points from ‘HTA expert commentaries’

These commentaries relay that it is time to put effort into the everyday experiences and issues that are being identified with HTA processes. HTA agencies need to be able to learn from practical experience of what works best for them [2, 5], and ‘imaginative innovation’ is called for [2, 5]. Each HTA agency has its own cultural, structural, and financial considerations, and each country has its own cultural, political and legal context for practising HTA [2]. It is important that HTA agencies are aligned with their environment, are socially responsible, and support healthcare systems in fulfilling their goals [5]. Goetghebeur and Cellier [5] ask if rigorous integration of qualitative research, including structured consultation of patients, citizens and relevant stakeholders, analyses of socio-political and the organizational contexts, and identification of ethical aspects across all the dimensions of evaluation could contribute to a better technology assessment.

### Examples of where changes are happening

Some HTA agencies have set up advisory or related groups involving patient advocates and patient group representatives. In Australia, the HTA Consumer Consultative Committee provides strategic advice and support to the main Australian HTA Committees and the Department of Health. This is related to ‘consumer engagement and participation in HTA processes’ [46]. The committee consists of ‘health consumer representatives’ who sit on the key HTA committees. One of the consumer representatives is Vice-Chair of PBAC [12]. In Scotland, the Public Involvement Network (PIN) helps to strengthen relationships with SMC and to in the development of new materials for patient groups. The objective is to continue to improve patient and public involvement. PIN is made up of ‘patient group partners’, representatives from three umbrella patient groups, SMC public partners and members of the SMC team, including a clinician from the SMC Committee [47]. At CADTH in Canada, the Patient and Community Advisory Committee (PCAC) is made up of individuals with lived experience of the Canadian healthcare system. This committee provides advice from the perspective of its members [48].

These examples show that HTA agencies are already making changes to provide patient ‘leaders’ and patient groups with advisory roles to improve patient input into HTA.

## Conclusions

In this commentary, we present a ‘Call to action’ for HTA agencies and stakeholders to work together to create a clear goal for meaningful patient involvement at every step of the HTA process across HTA agencies worldwide. To support this ‘Call to action’, we provide points of discussion on HTA processes and the call for learning and innovative ideas to create change. These were raised in three commentaries written by people well known in the area of HTA. Examples of where HTA agencies and others have introduced changes to HTA practices are given to stimulate action.

Five initial goals are defined to guide dialogue.

Under the first goal, we have highlighted values and quality criteria for ‘working together with shared purpose’. We give examples of patients with experiential and contextual knowledge working in partnership with medical practitioners and HTA staff. These have led to development of processes for defensible decision making in HTA and in providing patient input that contributes to HTA appraisal and decision making.

The second goal is for ‘a change in HTA culture, with integration of patient involvement’. Under this goal, the ‘expert commentaries’ raised many questions that are important to address on how patients could be involved and why. These could be used to path the way for a ‘change in culture’. We have highlighted examples from two HTA agencies where discussions have been initiated to address changes in culture to be more inclusive of patients.

The third goal, ‘alignment with HTA agency goals – and a positive impact on the diverse populations served’, highlights that Belgium and New Zealand have updated their HTA processes in consideration of patient perspectives. They now place a greater emphasis on quality of life of individual patients rather than life expectancy and cost-effectiveness. This can be seen as a move to achieve better health outcomes and therefore the stated goals of a number of HTA agencies, along with equitable distribution of technologies. It is important that agencies are able to demonstrate their impact on health systems and the value they add.

Under the fourth goal of ‘patient involvement at every step of the HTA process’, we have highlighted that patients can have a role in HTA processes from topic selection, scoping, examining the evidence, deliberations of appraisal committees to determine value, and in formulating recommendations for funding or subsidy. We have provided two examples of where HTA agencies have successfully utilised patients early in the process in order to broaden the scope of a report or evaluation. We need to involve patient advocates when examining what is the ‘best or most appropriate evidence’ for different HTA decisions. Early involvement in the design of clinical trials that provide clinical evidence on new technologies is also important, in methodological considerations such as the populations studied and to ensure that patient relevant outcomes are available, to inform HTAs.

As part of the fifth goal, ‘transformative thinking that involves patient leaders, with use of a unifying language’, patient advocates ask that HTA staff, practitioners and stakeholders work together to re-examine the objectives, roles and current tools and processes for patient and carer involvement in HTA. Integrating objective evidence with the findings from the lived experience and context provided by patients and their carers remains a challenge, but can still contribute to the relevance of HTA recommendations HTA agencies are already making changes to provide patient ‘leaders’ and patient groups with advisory roles to improve patient involvement in HTA.

We can explore that lived experience can be valid evidence within the right parameters and with a willingness to do so. That concepts such as ‘patient leadership’ can help systems understand how to invest in active, meaningful patient engagement and involvement. A unifying language could help, and seeing ‘participation as a science’ might legitimise patient involvement in the eyes of HTA practitioners. Capturing everyday experiences from a global environment can help to throw light on problems and lead to guidance on how to achieve identified goals of patient involvement.

The authors of this commentary consider that all involved in HTA (e.g. agencies, researchers, appraisal committees, industry, patient advocates) should take up this call and work together to elevate the voice of patients in HTA worldwide.

## Data Availability

A report of the 2019 HTAi PCIG workshop and review of the current literature is available from the corresponding author.

## Abbreviations

ACE: Agency for care effectiveness, Singapore
CADTH: Canadian agency for drugs and technology in health
CIUSSS de l’Estrie-CHUS: Centre intégré universitaire de santé et services sociaux de l’Estrie - centre hospitalier universitaire de Sherbrooke (CHUS), Quebec, Canada
EUnetHTA: European network for health technology assessment
FDA: Food and drug administration, United States of America
HITAP: Health intervention and technology assessment program, Thailand
HTA: Health technology assessment
HTAi: Health technology assessment international
ICER: Institute for clinical and economic review
INESSS: Institut national d’excellence en santé et en services sociaux, Quebec
KCE: Belgium health care knowledge center
NHS: National health service
NICE: The national institute for health and care excellence, England
OPP: Operating policies and procedures
PBAC: Pharmaceutical benefits advisory committee, Australia
PCIG: HTAi patient and citizen involvement in HTA interest group
PHARMAC: Pharmaceutical management agency, New Zealand
RIZIV/INAMI: National institute for health insurance, Belgium
SMC: Scottish medicines consortium
